# Supplementary material for: An exploratory Study of EEG Alpha Oscillation and Pupil Dilation in Hearing-Aid Users During Effortful listening to Continuous Speech
Source: PLoS One. 2020 Jul 10;15(7):e0235782. doi: 10.1371/journal.pone.0235782 (PMC7351195; doi:10.1371/journal.pone.0235782)
Supplement: Attachment — Submitted filename: Response to Reviewers.pdf [file pone.0235782.s001.pdf]

Dear Profs. Lee and Winn,

Thank you both for your valuable comments on the manuscript. We greatly appreciate your time and consideration, which has helped us to vastly improve the manuscript.

We have addressed each of the points you have raised with amendments and additional analyses to the manuscript as detailed below. Of particular note, we have addressed the collective concern over the low *n* by highlighting the exploratory nature of the study – and its limitations – throughout, from the title to the conclusions, scaling back claims to be commensurate with the study's scale. We have also addressed the questions raised by both of you over the spatial nature of the task by clarifying the method much more thoroughly, and reporting additional analyses as suggested by Prof. Lee. We are particularly appreciative of this suggestion, along with the additional time-window analysis suggested by Prof. Winn, as these new results pose questions (e.g., does alpha lateralization only occur in quiet?) that give the manuscript much greater import for the readers of PLoS One.

Please note all the comments from the reviewers are in **bold**, responses are indented, and all changes to the manuscript are double-indented in *blue italics*.

Best regards,

Tirdad Seifi Ala

Reviewer #1

This paper describes a study where listening effort was measured in response to continuous speech passages. The major novelty of the study is that the stimuli are longer than what is typically used in most studies of effort, which usually use single sentences. The current experiment used listeners with hearing loss who were fit with hearing aids. The data suggest that pupil dilation declines over the course of prolonged listening, but declines at a slower rate (i.e. remains more dilated) when the speech is more difficult. The EEG results were less clear, and there was no correlation between the two measurements on a global level.

The study offers a potentially valuable contribution to the literature on listening effort. The major flaw – the number of participants is addressable without changing the design of the study. The other limitations of the manuscript itself are also addressable. I will outline the major points below, as well as some minor things that could improve the paper. At the moment, I do not think the paper is worth reviewing again without additional data collection from a larger number of participants.

Major comments

1) Number of participants. The number of participants in the study is too small. The authors attempt to soften this limitation by explaining in line 376 that individual data are rather consistent in the pattern across conditions. This is mostly true for the pupil data and not for the EEG data. Regardless, it appears to me as if the study is simply not complete. Why stop at 8 participants? As the main motivation for the study appears to be the establishment of the method of measuring responses to continuous speech rather than the use of listeners with hearing aids, it would seem feasible to add power to the study by having more easily accessible sample of listeners with typical hearing. If the goal is to learn something about hearing impairment/the use of hearing aids, there is just too small a number of listeners.

The reviewer is entirely correct that this was a small-scale, exploratory study, which we did not adequately highlight in the original submission. Unfortunately, as the original data was collected more than a year ago there are several reasons that collecting more data now is not possible: (a) in the intervening time the lab and its setup have undergone a lot of changes, (b) the first author has moved to a different institution in a different country, and (c) the current global situation makes it unlikely it would be possible to run any participants in the foreseeable future. While any one of these conditions would not be reason enough to not continue the study, the combination of all three makes it much more difficult to add data now or in the future.

To better acknowledge this limitation in the paper, we have worked to improve the transparency of this issue throughout. It also is now emphasized from the outset in the title:

An Exploratory Study of EEG Alpha Oscillation and Pupil Dilation in Hearing-Aid Users During Effortful listening to Continuous Speech

And we have also made sure to reference the scale of the study throughout the manuscript and appropriately hedge our findings:

Eight hearing-aid users participated in this exploratory study and performed a continuous speech-in-noise task. [Lines 24-25]

To begin investigating physiological changes during a listening task in more ecologically valid situations, we conducted an exploratory study where continuous auditory news clips were presented to hearing-impaired participants at two different SNRs. [Lines 124-127]

In this exploratory study pupillometry and EEG were used to assess listening effort of hearing-aid users in a continuous speech setting. [Lines 481-482]

Additionally, we have pointed out this limitation and tempered how the results can be considered:

The second limitation is the low number of participants (n = 8) recruited for this experiment. Although this affects the statistical validation, the normality assumption of the data was checked by both Kolmogorov-Smirnov and Q-Q plot. Also, as an initial investigation into these physiological measures in a continuous task, we aimed to rely less on interpreting the p-values and more on the high consistency of individual responses by providing single-subject results (right panels in Fig. 2). [Lines 466-471]

We did not include the K-S test results and Q-Q plots in the text, as we feel it would deter emphasis away from the results, but provide them below:

df = 96, p = .070 | df = 96, p = .109

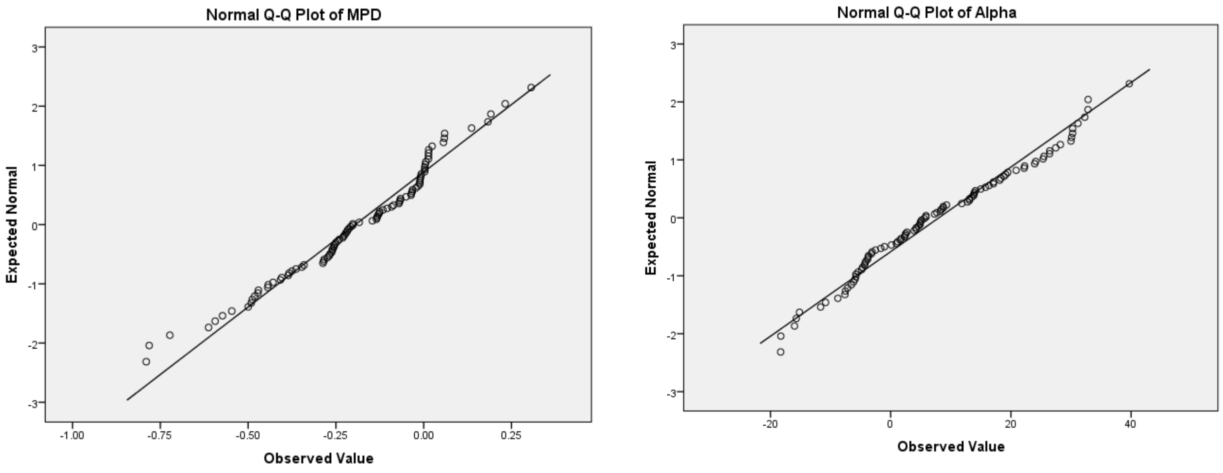

Fig 1. *Q-Q plots for **Left)** MPD and **Right)** Alpha power. The p-values show the results of Kolmogorov-Smirnov test.*

2) Conclusion: "This study demonstrates that pupillometry and EEG can be effectively used to assess listening effort" This seems premature to me. I don't see how the authors justify calling this method "effective", or how they have established that the changes in EEG/pupil size index the same thing. I would recommend rewriting the conclusions to convey only what is clearly indicated by the data, and leave speculation in the discussion section. As you might guess, I think that part of why this conclusion is premature is because the number of participants is too small. To validate this new approach to measuring effort for continuous speech, you really ought to make sure you understand the extent of individual variability and establish that it isn't just something that explains these eight people.

Corrected. The conclusion began far too speculative, and has been rewritten to be clearer on the data and its limitations:

> *In this exploratory study pupillometry and EEG were used to assess aspects of listening effort of hearing-aid users in a continuous speech setting.* *[Lines 481-482]*

Please also see the changes made above with respect to the small number of participants in this admittedly exploratory study, and our new handling of individual variability.

3) Methods – please explain the physical location of the maskers - why was there a masker 60 degrees lateral of the target and also one masker behind the listener? What was the rationale for having a slightly-left or slightly-right location of the talker, and for the target location to randomly alternate between trials? For a study that emphasizes the need to incorporate ecological validity, this seems like an odd setup that is perhaps contrived to exploit specific kinds of hearing aid processing.

Thank you for pointing out this lack of clarity. We have revised the Methods in several places as detailed below to be clearer about the locations and their roles.

The rationale behind choosing the set-up was not to exploit hearing aid processing, most of which was deactivated – which is now clearly stated:

> *Noise reduction and directional microphones were deactivated so that the hearing aids just provided individualized audibility via the proprietary gain and frequency prescription rule.* *[Lines 147-149]*

The rationale was to create an admittedly modestly ecological scenario, where there are two competing talkers with some spatial separation. The reason to alternate between right and left target was (a) to prevent any minor asymmetrical hearing loss influencing the results:

> *The loudspeakers in the front hemifield were the target and distractor locations, symmetrically off-center to counterbalance any asymmetrical hearing abilities, and the loudspeaker in the rear hemifield presented 4-talker babble noise to increase task complexity.* *[Lines 153-156]*

and (b) to provide comparison with previous studies of alpha lateralization:

> *These contradictory results show the ambiguity of interpreting alpha power changes in listening, as listening can involve different cortical processes, depending on the speech material or its presentation (Peelle, 2012). For example, Wöstmann et al., 2016 and Deng et al., 2020 have shown differences in alpha lateralization when presenting competing speech from contralateral locations.* *[Lines 116-120]*

While the notion of 4-talker babble coming from one location is admittedly hardly ecological, it increases the complexity of the situation in line with a more ecological situation. The background noise also provided a distinction from previous alpha lateralization studies. In coordination with the comments from Prof. Lee, we now highlight this distinction and its import towards future work:

The spatial setup with a contralateral distractor in this study provided the chance to look at the alpha lateralization in a more realistic situation with background noise. However, unlike previous studies (Wöstmann et al., 2016; Deng et al. 2020), no difference between hemispheres was observed in the data. One key difference between those previous studies and the current study is the addition here of four-talker babble noise from directly behind the listener. The presence and/or location of the background noise in the current study may have obscured any indication of alpha lateralization. Another difference between the current and previous studies is that listeners were bilaterally aided, which may have also affected alpha lateralization. Further studies are required to fully explore this lack of alpha lateralization, but this result highlights the potential importance of using a background noise in spatial attention tasks. [Lines 413-423]

4) Review of literature
The review of literature should actually inform the reviewer of how some of the critical knowledge was acquired rather than just make statements followed by names and years. In general, there are very many places in the paper where the literature review is not helpful, because it either 1-makes statements without any explanation of how the knowledge was acquired, or 2-conflates a hypothesis with fact. I will give some examples:

Line 48-49: "Even if individuals can hear what is being said, they may need to put more effort in to process the auditory input (Lunner et al., 2016)."

Tell us how this is known. As it is written, you're just asking the reader to trust Lunner that this is true. How was this conclusion established? There are lots and lots of published papers that have established this kind of conclusion using various methods. It would be an incomplete paper without actually describing the work that has come before. We can't just make statements and attach names to them as if the name alone provides the authority to trust the claim. Tell us that "Author A did a study where B was measured using C as a stimulus. Group D performed differently than group E, allowing us to conclude F." This is much more informative than "F is true (Author A)."

We have gone through the Introduction and revised any and all instances (incl. the egregious one highlighted above) of non-logical referencing. As an example, the above has been properly detailed as advised:

The second issue is that even if speech intelligibility is optimal, other cognitive factors might be changing with the difficulty of the task. For example, Sarampalis et al., 2009 showed that using a noise reduction scheme in hearing aids did not improve intelligibility but did improve performance in a simultaneous visual task. Houben et al. (2013) showed when the speech intelligibility is at ceiling, increasing the signal-to-noise ratio (SNR), reduced the response time of a simultaneous arithmetic task. Both studies concluded that reducing the difficulty of the speech task reduces the cognitive demand which leads to a reduction in listening effort. [Lines 64-71]

Line 43-44: "free-running, connected discourse, that mainly exists in real life situations, can hardly be analyzed by word or sentence intelligibility (Speaks et al., 1972)"
What is meant by this? Why can't it be analyzed that way? Tell the reader why this is the case. If you delete the sentence that begins with "Henceforth", then it could be clearer.

Corrected. The "Henceforth" sentence has been deleted as suggested, and the sentence in question was misleading in mentioning analysis, and has been revised:

> *The first issue is that in real life, most listening situations involve conversations with free-running, continuous discourse, and do not stop after every few words (Speaks et al., 1972; MacPherson and Akeroyd, 2013). [Lines 62-64]*

Line 355: "…the frontal theta showed an increase in more demanding situations"
What is meant by "more demanding situations"? This is another one of many examples in this paper where the literature is not reviewed in a way that allows the reader to understand what work was actually done. Same for the following line where it is not clear what is meant by "a linguistic task".

Both corrected. In the first study we added that by "more demanding" we meant introducing a *retention phase to the task* and in the second study by "linguistic" we meant *disyllabic word recognition*:

> *For example, when the participants were asked to recognize the highest pitch when exposed to square waves, the frontal theta showed an increase in more demanding situation where retention was required to perform the task (Wisniewski et al. (2018)). On the other hand in a speech-related task, Marsella et al. (2017) demonstrated that degrading the SNR in a linguistic task consisted of disyllabic words in children with asymmetric sensorineural hearing loss did not result in higher frontal theta activation. [Lines 445-450]*

Line 36: "helping to reduce these limitations in everyday life" – do these studies actually demonstrate that hearing aids lead to reduced fatigue, increased social participation and engagement in conversations and improve recall? I suspect that these things have not actually been established, and it is important to recognize what is "hypothesis" and what has actually been empirically demonstrated.

That phrase is indeed a gross overstatement, and has been couched as hypothetical potential, and appropriate citation of particular instances of benefit relevant to the current study:

> *Hearing devices can assist those with a hearing loss, and may help to reduce some of these limitations by improving memory (Ng et al., 2015), reducing listening effort (Ohlenforst et al., 2018) and response time (Gatehouse and Gordon, 1990), as well as providing long-term benefits such as social and emotional improvement (Mulrow et al., 1992). [Lines 47-50]*

Line 34: "recalling the speech" – is the Rönnberg study an empirical study demonstrating this, or a theory paper? Studies on recognition memory/recall have been done by Smiljanic, Van Engen, Gilbert, and others.

Corrected. Revised to reference the suggested studies by *Van Engen et al. 2012* and *Ward et al., 2016* as well as provide the necessary detail which are empirical demonstration of speech recall.

> *These issues in speech recognition can cause excessive cognitive load, which can in turn lead to negative effects such as difficulties in comprehension (Wingfield et al., 2006), recalling the speech (van Engen et al., 2012; Ward et al., 2016), fatigue (Wang et al., 2018) or disengagement from conversations (Jaworski and Stephens, 1998). [Lines 41-45]*

Starting at line 300: the discussion of studies by Peterson and by Miles is not as clear as it could be. The specific stimulus that these studies used is very important. The manuscript says that Peterson used monosyllabic digits – this is helpful because it means that there was almost no language processing like syntactic or semantic context processing. No mention was made of the stimuli used by Miles, which makes this section incomplete. Is it possible that the difference between these contradictory studies simply reflects the different demands of the stimuli used?

Corrected. We added that study by Miles is *speech recognition task* (as opposed to digit recognition to in Petersen). Please note the conclusion in the end agrees with you that different stimuli require different demands and different processing and that's why EEG results are contradictory.

> *On the other hand, Miles et al., 2017 reported that in a speech recognition task, parietal alpha was decreased during a demanding situation when the spectral content of the signal using noise vocoding and speech intelligibility were changed. Though clearly a disputed concept, listening effort related changes in alpha power are probably a function of the speech material used and may vary based on the definition of the "listening" task and/or different demands which require top-down or bottom-up processing. [Lines 380-386]*

Line 325-333: the discussion of the retention interval in EEG studies is good, but it highlights the absence of a similar discussion of retention interval in pupillometry studies, e.g. by Piquado et al., and by Winn & Moore.

Excellent point; we have added a whole new paragraph in the pupillometry discussion to talk about this point.

> *In addition to pupil changes during listening, studies (e.g., Piquado et al., 2010; Winn and Moore, 2018) have shown pupil size can also change after the listening phase and during retention. Sustained increase of pupil dilation in the retention phase can happen in more demanding conditions. While in this study there is no retention phase, it can be argued that presenting long, continuous-speech stimuli requires gradual retention, especially towards the later parts of the stimulus, which can affect pupil dilation. [Lines 366-371]*

5) Figure 2 – the design is very nice, and easy to understand. But there are two things to change: first, do not use a red & green color pattern, because a common form of colorblindness means that readers cannot distinguish these colors. Any other color pair would be better. This goes for both the EEG data, pupil data, and the brain topomaps. Second, please produce this figure with higher resolution, as it appears very blurry.

Corrected as advised; we have changed colors from green and red, with higher resolution and more succinct (combining Figure 2 and 3 in the previous manuscript together in the current version as panels in Figure 2).

6) The title of the paper is entirely uninformative. The title should indicate something about what was found. As it is currently written, it is more like "click bait"

Apologies, we never intended to make anything remotely like click bait, but now realize it is far from ideal. In line with the initial comment, we have changed the title to be more descriptive and tempered in terms of the scale of the study:

> *An Exploratory Study of EEG Alpha Oscillation and Pupil Dilation in Hearing-Aid Users During Effortful listening to Continuous Speech*

Minor comments

Line 45: improper use of the word "henceforth" - that would mean "from this point forward…"

Corrected; we have removed that sentence in line with a previous comment.

Abstract: the sentence "This sustained attention requires cognitive resources, the expenditure of which leads to listening effort." Could be removed, as it is reasonable to rephrase as "X requires X, leading to X", where X is the same concept repeated multiple times redundantly, just with different words.

The abstract is misleading when it implies that pupillometry is a well-established method to look at sustained attention or effort. It is in fact a well established way of looking at momentary, evoked or episodic effort, not sustained effort. But I think this is actually the true value of the study – that the authors are looking at continuous speech rather than single utterances. As it is currently written, it looks like the paper is framing the main contribution as the combination of EEG and pupillometry, which is not novel. I would suggest emphasizing that the main contribution is the transition from single utterance to continuous speech.

We definitely agree with you that the novelty of the paper is using these methods in a continuous speech rather than combination of the two measures. We have changed the phrases in the abstract with your suggestion to the following:

> *Such a scenario could be when they are exposed to ongoing speech and need to sustain their attention for a rather long period of time, which requires listening effort. Two well-established physiological methods that have been found to be sensitive to identify changes in listening effort are pupillometry and electroencephalography (EEG). However, these measurements have been used mainly for momentary, evoked or episodic effort. The aim of this study was to investigate how sustained effort manifests in pupillometry and EEG, using continuous speech with varying signal-to-noise ratio (SNR).* [Lines 16-25]

Abstract: "The effects of SNR on listening effort were explored objectively using pupillometry and EEG data."
Omit the word "data" because the exploration was done using the method, and the data resulted from the method; the data are not the method.

Corrected. Removed.

Line 29: "interpreting" – this should be "perceiving", as "interpreting" could imply that what you're talking about is the translation of spoken language to sign language.

Corrected. Replaced.

Line 53: "To assess listening effort, it is necessary to measure it objectively by monitoring the changes that occur in the central and autonomic nervous systems during speech processing"
This is an opinion, and it is generally thought to be false; one can also measure listening effort using measures other than physiological methods. For example, people can measure reaction time, dual-task cost, subjective measures, etc. It is not *necessary* to objectively monitor autonomic changes.

We completely agree, the sentence was not worded correctly, and we have revised thoroughly:

> *There are myriad ways to assess listening effort (Alhanbali et al., 2019): self-report, behavioral responses such as reaction time (e.g., Sarampalis et al., 2009) or by monitoring the changes that occur in the central and autonomic nervous systems during and after speech processing (e.g., Obleser et al., 2012; Rudner and Lunner, 2014).* [Lines 81-84]

Line 193: "The bias filter in this method for denoising was chosen as the average of trials"
I do not know what this sentence means. Please explain.

We have added an explanation of the role of the bias filter:

> *The bias filter in this method for denoising was chosen as the average of trials. Such a bias filter enhances the optimal weights for independent components in a way that components have the most repeatability across all trials.* [Lines 240-241]

Line 288: "The relative decrease of MPD measurement over 30 seconds might emphasize the sustained attention of the task at hand, which, as a result, led to listening effort"

I don't understand the logic of this sentence. I don't see how the decreased pupil size can "emphasize" sustained attention, and I do not understand what the authors are saying led to listening effort. Please explain.

Corrected. The negative MPD is probably reflecting the evoked response in the baseline when subjects are exposed to background noise.

> *The relative decrease of MPD measurement over 30 seconds might be due to evoked pupil dilation to the background noise in the baseline. Nevertheless, it is clear from Fig. 2C that in the harder condition, MPD was still higher (less negative) for continuous speech, which demonstrates increased listening effort for sustaining attention. Larger pupil dilation during demanding conditions has been associated with increased workload and a greater allocation of resources to perform the listening task (Wendt et al., 2017).* [Lines 359-365]

Since the stimulus/task is so prolonged compared to previous pupillometry studies, it's not clear to me why the authors didn't devise a new metric that is actually suited to this design. It looks like measurements suitable for short-stimulus design were used here without much reflection on whether they were appropriate. As the use of continuous signals is rather novel for this study, it is a missed

opportunity for the authors to offer a new kind of analysis that is actually designed for this procedure. Slope measurements seem like a reasonable start?

Thanks for the excellent idea.

First, we have acknowledged that because it is a long stimulus, we have used mean pupil dilation instead of peak pupil dilation (which is more suitable for short stimuli):

> *MPD was applied since it is more robust compared to PPD in longer stimuli designs, as MPD extracts all the information within 30 seconds of data. In contrary, PPD usually happens only in the first few seconds of the target onset and gives no further information for the rest of the stimuli.* [Lines 221-224]

Also one other reason that we chose mean pupil dilation was that the data points in pupillometry can be aligned with the ones in EEG (5 sec) which gives us the same time data points for analysis of covariance in both measurements.

To add a slope analysis as suggested, we have added an analysis of difference between the time-windowed mean pupils. In the *Methods (Pupillometry)* we acknowledge longer stimuli provides more feature extraction possibilities:

> *The longer stimuli also provide the opportunity for exploring other features within pupil data such as the difference in the MPD. For this reason, the difference of time-windowed mean pupils was compared between low vs. high SNR.* [Lines 225-227]

And as a result, it also has been added to the *Methods (Statistics)*:

> *For MPD, difference in MPD, theta power and alpha power repeated measure ANCOVA was used, with SNR as the predictor and Time (0-5, 5-10, 10-15, 15-20, 20-25, 25-30 sec.) as the covariant factor.* [Lines 275-278]

However, in the *Results* it has been mentioned that it did not show any significant difference between SNRs:

> *The comparison between the difference in MPD showed no significant change between low vs. high SNR [F(1,46) = 2.685, p = 0.108] nor an interaction between SNR and Time [F(1,46) = 2.132, p = 0.151]. These results show that in longer stimuli mean pupil is still more sensitive to task demand than its changes.* [Lines 300-304]

Line 335: "alignment" is a confusing word here, since it implies a correlation that is later said to be absent. The authors of that work instead used the word "coregistration", conveying the idea that the two measurements were made at the same time but does not promise a correlation.

Corrected to *co-registration*.

Line 343: This section is good, since it reminds the reader that lack of correlation between EEG and pupil measures is not necessarily a problem, but perhaps a sign that they index different brain functions. However, there is a flaw in the argumentation. In line 343: "Pupil diameter is suggested to reflect locus coeruleus–noradrenergic (LC-NE) neuro-modulatory system which increases task relevant neuronal gain in cerebral cortex (Murphy et al., 2014)." While it is not controversial to connect pupil dilation to the LC, it has also been established that pupil size correlates with a broad range of distributed cortical activity (Reimer et al., 2016). In other words, just because pupil size correlates with LC activity, that doesn't mean it correlates *specifically* with LC activity.

Thank you for this important point and recommendation of this very relevant paper. We have added this important distinction as suggested:

Pupil diameter is suggested to reflect different neuro-modulatory systems such as locus coeruleus–noradrenergic (LC-NE) which increases task-relevant neuronal gain in cerebral cortex in rapid dilations (Murphy et al., 2014) or basal forebrain which modulates the state of cortical activity during sustained activity (Reimer et al., 2016). [Lines 433-436]

Line 351: "The theta band, which oscillates in slower frequencies than alpha band, has been widely recognized as "cognitive effort" in many non-auditory working memory tasks"

I think there's a word or two missing here – the theta band itself isn't recognized AS effort – perhaps it is recognized as reflecting effort or indicating effort?

Apologies, there are indeed several words missing, we have now corrected the sentence to clarify:

The theta band, which oscillates in slower frequencies than alpha band, has been widely recognized as neural correlates of "cognitive effort" in many non-auditory working memory tasks. [Lines 442-443]

Acknowledgments - One Polish character is used properly in "Skłodowska" but other Polish letters were missing from "Książek". This is nitpicky, but since it involves a person's name I thought it would be worth correcting.

Corrected; we are embarrassed to have misspelled one of our colleagues.

Figure 4 – this figure is difficult to read and also lacks regression lines among data subsets (i.e. between all the 0-5 timepoints, between all the 5-10 timepoints, etc.) in addition to the overall regression line. Perhaps there is a positive correlation between 10 and 15 seconds?
The color scale in Figure 4 is also quite unhelpful because there is no orderly manner to view the differences across time. If the color saturation increased / got darker with increasing time, then it would be easy to follow across the different time points. But as it's drawn now, it's not easy to see why the different timepoints are even shown, because the reader can't possibly separate them or follow them along.

We have added as suggested regression lines (dashed) for each data point with the same colors to help readers to follow them better in the Figure (Fig. 3 in the new manuscript).

We attempted to use a gradient for each timepoint, but found it to be less readable in grayscale. Instead we have changed the colors of the individual points to hopefully create a better contrast for the reader.

Reviewer #2

The authors investigated listening effort of individuals with hearing loss using both pupillometry and electroencephalography (EEG). The main findings are that the overall parietal alpha power obtained in EEG and the mean pupillometry diameter dilation both vary with target and masker ratio (referred to as SNR in this manuscript), suggesting that both measures are sensitive to listening effort, consistent with how other authors interpret these findings in the literature.

The study is generally well conceived, described and executed (albeit with a relatively small N, but provided the necessary caveats). I only have one major suggestion and some minor editorial points.

Major suggestion

Since this is a spatial attention task, the parietal alpha power might be modulated differently across hemispheres depending on the side of attention (e.g., Deng, Reinhart, Choi and Shine-Cunningham, eLife 2019). I suggest the authors reanalyze the parietal alpha power by binning the left target and the right target speaker separately. This may provide more signal to the study.

Thank you for raising this point. With the spatial setup in our design, it is an excellent opportunity to look for alpha lateralization for any evidence of different spatial attention processing in the brain. For this purpose, we looked at the difference between alpha power in “attend right” and “attend left” situations and compared the right hemisphere of the brain to the left hemispheres.

We have added this analysis to all sections of the paper, as detailed below. We did not include the figures in the text, as we feel it might not add additional information to the reader given the lack of differences found, but provide them below:

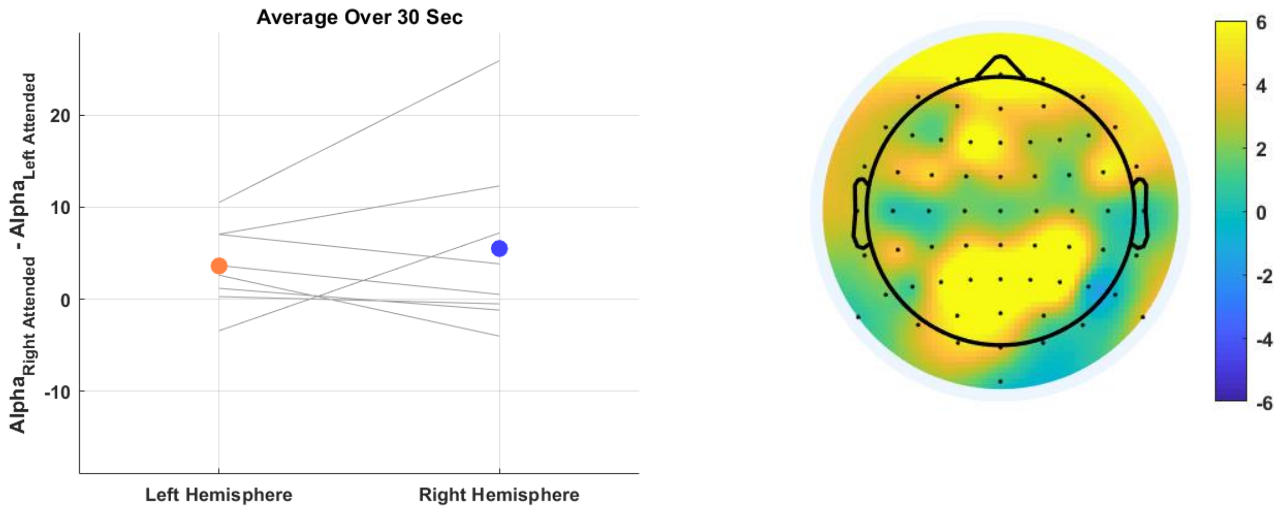

Fig 2. *Assesment of AMI:* ***Left)*** *Comparison of left hemisphere vs. right hemisphere. No statistical difference between the two. The average results and each indiviual subject are presented.* ***Right)*** *Topographic maps for Alpha_Right – Alpha_Left measurement.*

Alpha lateralization added to the *Introduction*:

> *These contradictory results show the ambiguity of interpreting alpha power changes in listening, as listening can involve different cortical processes, depending on the speech material or its presentation (Peelle, 2012). For example, Wöstmann et al., 2016 and Deng et al., 2020 have shown differences in alpha lateralization when presenting competing speech from contralateral locations. [Lines 116-120]*

In the method (*Alpha lateralization*)

> *Alpha lateralization was also investigated to see if attending right vs. left stimuli elicits different responses in different hemispheres. To do so, the alpha power when the participants were attending to the right target was subtracted from the alpha power when the participants were attending to the left target for both lateral hemispheres. Then, right hemisphere was compared to left hemisphere to see if they respond differently, depending on the location of the target. [Lines 266-271]*

The statistical approach for alpha lateralization has been added to mentioned in *Statistics*.

> *For alpha power lateralization, the same Time windows as covariant factors were used, but unlike other dependent variables, right vs. left hemispheres were used as predictors of the repeated measure test. [Lines 278-281]*

In the results (*Alpha lateralization*):

> *For alpha lateralization, the difference between "attended right" and "attended left" in the right and left hemispheres did not show any significant difference [$F(1,46) = 0.049$, $p =$*

0.826], suggesting the location of the target did not elicit different responses between hemispheres. [Lines 336-339]

In the *Discussion* we consider why the alpha lateralization did not happen in our study, which is speculative but might raise several research questions in future:

The spatial setup with a contralateral distractor in this study provided the chance to look at the alpha lateralization in a more realistic situation with background noise. However, unlike previous studies (Wöstmann et al., 2016; Deng et al. 2020), no difference between hemispheres was observed in the data. One key difference between those previous studies and the current study is the addition here of four-talker babble noise at 50 dB from directly behind the listener. The presence and/or location of the background noise in the current study may have obscured any indication of alpha lateralization. Another difference between the current and previous studies is that listeners in the current study were bilaterally aided, which may have also affected alpha lateralization. Further studies are required to fully explore this lack of alpha lateralization, but this result highlights the potential importance of using a background noise in spatial attention tasks. [Lines 413-423]

Minor suggestions

Ln 58: change "to measure electoral activity caused by neural oscillations" to "measure neural oscillations"

Corrected. The term is now replaced with “measure neural oscillations”.

Ln 154: the reader might want to know what type of questions are being asked and what type of answers are provided in the 3-AFC behavioral measurement. Perhaps you can provide a typical question (translated) so that the readers get a sense of the scope of the question (and the level of cognitive process that might involve in getting them right)?

We added an example question to the text as suggested:

The target and distractor speech were presented 5 seconds after the onset of the babble (i.e., after the baseline period) and then continued for 30 seconds, followed by a three-choice question regarding the content of the attended target audio clip [e.g., “Who warns against the dangers of discrimination?” (English translation)]. [Lines 197-198]

Ln 255: change "showed less activation" to "was decreased"

Corrected.

Ln 257: change "topomap" to "topographical map"

Corrected, and have also changed in the caption of figure 2A.
